# Vines avoid coiling around neighbouring plants infested by polyphagous mites

**DOI:** 10.1038/s41598-019-43101-0

**Published:** 2019-04-29

**Authors:** Tomoya Nakai, Shuichi Yano

**Affiliations:** Laboratory of Ecological Information, Graduate School of Agriculture, Kyoto University, Sakyo-ku, Kyoto 606-8502 Japan

**Keywords:** Agroecology, Behavioural ecology

## Abstract

Vines that coil around plants heavily infested with ambulate polyphagous mites can be heavily damaged by the mites. To explore whether vines avoid mite-infested plants, we observed the coiling responses of morning glory (*Ipomoea nil* var. Heavenly Blue) vines and bush killer (*Cayratia japonica* (Thunb) Gagnep) tendrils around nearby kidney bean (*Phaseolus vulgaris* L.) plants that were either uninfested or heavily infested with the two-spotted spider mite (*Tetranychus urticae* Koch). The proportions of *I*. *nil* vines that coiled around spider mite-infested and uninfested bean plants did not differ significantly; however, no *C*. *japonica* tendril coiled around spider mite-infested plants. The proportion of such tendrils was thus significantly lower than that around uninfested plants. The ability of *C*. *japonica* tendrils to avoid spider mite-infested plants would prevent serious “contact infections” by mites. We further found that tendril avoidance seemed to be attributable to the mite webs that covered infested plants; neither spider mite-induced bean volatiles nor spider mite intrusion onto tendrils seemed to explain the avoidance.

## Introduction

Vines coil around neighbouring plants, seeking support^1^. This may allow small ambulate herbivores living on the support plants to invade the vines. In particular, physical contact between vines and their neighbouring plants mediates vine invasion by small wingless arthropods such as spider mites.

Spider mites (*Tetranychus urticae*) often exhaust the resources of the host plant, and mated adult females thus seek to disperse to new hosts (primarily via walking)^2^. Ambulatory adult female spider mites follow trails established by preceding females, triggering aggregations at new colonisation sites^3^. Spider mites can be found on hundreds of host plants from many families^4^; the mite is thus likely herbivorous on both vines and the plants around which vines coil. Therefore, vines coiling around plants heavily infested by spider mites will be infested by mites entering from the support plants. Since plants that can prevent feeding damage by all means are always advantaged, we predicted that vines would not coil around plants infested by spider mites.

Some parasitic plants use volatile cues to locate hosts^5^. The tendrils of the common wild vine *Cayratia japonica* (Thunb) Gagnep (Vitaceae) avoid harmful interactions with vines of both the same and related individuals^6^. The avoidance behaviour of *C*. *japonica* tendrils is thought to feature the chemoreceptive detection of chemical cues^7^. Given such sophistication, we hypothesised that *C*. *japonica* tendrils, and those of other vines, might not coil around plants infested by spider mites.

Spider mite-infested plants exhibit two specific changes. First, the plant surfaces become covered with mite webs, under which the mites feed and reproduce^8^. Second, the infected plants produce mite-specific volatile compounds^9^. Both changes are reliable indicators of a mite infection and are used by predators to detect the mites^10–12^. We hypothesised that such changes might be utilised by vines to detect spider mites on adjacent plants (hereinafter, “contact plants”).

We explored whether *C*. *japonica* tendrils avoided coiling around spider mite–infested bean plants. For comparison, we also examined vines of a common cultivated plant (*I. nil*). The effects of mite webs and mite-induced plant volatiles (hereinafter, SMIPVs) on the avoidance responses of vines adjacent to mite-infested plants were examined.

## Materials and Methods

### Plants and animals

*Ipomoea nil* is an annual vine often cultivated to form green curtains. Plants were individually grown in 200-mL plastic pots containing commercial soil (Tachikawa Heiwa Nouen, Kanuma, Japan) at 25 °C and 50% relative humidity under a 3,000-lux photoperiod (16 h on/8 h off).

Bush killer (*C*. *japonica*) is a common perennial vine that reproduces via both seed production and vegetatively. Both *C*. *japonica* and neighbouring plants are often infested with *Tetranychus* spider mites^13,14^. Study plants were collected from May to November in 2016 on the campus of Kyoto University, individually transplanted into 500-mL plastic pots filled with soil, and grown in a laboratory for >1 month. To allow free tendril movement, the main stems were supported with wires when necessary.

The target plant (in terms of coiling by *I*. *nil* and *C*. *japonica*) was the kidney bean (*Phaseolus vulgaris* L.). The plants were grown in plastic pots (12 cm in diameter, 10 cm in depth) filled with Akadama soil (Keiyo, Chiba, Japan) in the laboratory. About 2 weeks after germination, plants with fully expanded true leaves were used in our experiments. *Tetranychus urticae* is a polyphagous spider mite that feeds on hundreds of host plants^4^, including *I*. *nil*, *C*. *japonica*, and *P*. *vulgaris*. We infected bean plants with spider mites that had been reared on such plants for more than 10 years in the laboratory.

### Coiling responses of *I*. *nil* and *C*. *japonica* to bean plants infested with spider mites

We observed the coiling responses of *I*. *nil* vines and *C*. *japonica* tendrils to bean plants either uninfested or heavily infested with spider mites. As both *I*. *nil* vines and *C*. *japonica* tendrils move in fixed directions at all times, a fair choice between two target plants is difficult because, when presented two plants, the first one contacted by a tested vine is always fixed. Therefore, we adopted non-choice experiments (coiling around either infested or uninfested plants) rather than dual choice experiments. To prepare the target plants, at least one expanded true leaf was cut from the major stem and wrapped in wet absorbent cotton. The cotton was inserted into a 10-mL glass tube to ensure that the cut plant remained perpendicular (uninfested plants). These plants were about 15 cm in height. Bean plants of the same age that were heavily infested with spider mites (>30 adult females and individuals at other stages per leaflet) were treated in the same manner (infested bean plants). The stems of the infested bean plants bore many mite webs.

**Figure 1:**
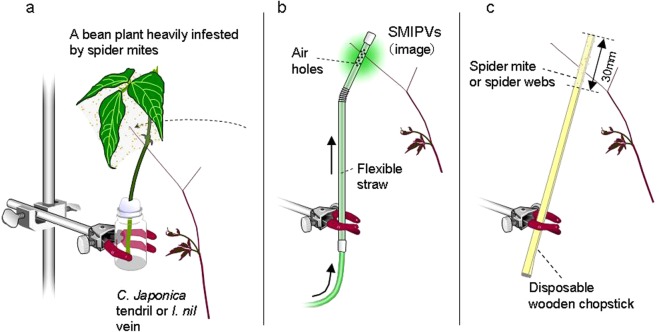
Experimental setups used to investigate (**a**) the coiling responses of *C. japonica* tendrils to spider mite-infested bean plants, and to investifate the effects of (**b**) SMIPVs and (**c**) spider mite webs on the coiling response of *C. japonica* tendrils.

To forecast *I*. *nil* circumnutation, the plants were subjected to time-lapse photography (1 frame/min) using a web camera (UCAM-CO220FBN; ELECOM, Osaka, Japan) and image editing software (Interval Shot; Department of ICT and Vocational Education, General Education Center of Iwate, Iwate, Japan). Only circumnutating *I*. *nil* plants >15 cm in height were used. We then clamped a bottle bearing either an infested or uninfested bean plant in the position of future *I*. *nil* circumnutation (Fig. 1a). Each *I*. *nil* plant was randomly placed near either an infested or an uninfested plant and was used only once. The base of the vine tip was grease-banded with Tanglefoot (Tangle B; Fuji Chemicals Industrial, Tokyo, Japan) to prevent any mass inflow of mites into *I*. *nil* leaves. To control for potential bias, *I*. *nil* plants placed near uninfested bean plants were treated in the same manner. Vines were considered to have coiled when they proceeded around the plants by >180°. Circumnutating *I*. *nil* vines that failed to reach bean plants either because of deviation from the predicted course or irregular movement of the bean plants were excluded from analysis. We then compared the proportions of vines coiled around infested (n = 17) and uninfested (n = 10) bean plants using Fisher’s exact test (SAS 9.22; SAS Institute, Cary, NC).

Tendril movement of *C. japonica* was forecasted using the methods described for *I*. *nil* above (Fig. 1a). Only plants >20 cm in height with active tendrils were used. The bases of all tendrils were grease-banded. Tendrils were considered to have coiled when they proceeded around the plants by >180°. Tendrils that failed to touch bean plants were excluded from analysis. Each *C*. *japonica* plant was used only once to preclude effects from previous experiments (Yano, unpublished data). We then compared the proportions of tendrils coiled around infested (n = 5) and uninfested (n = 12) bean plants in the manner described above. As infested bean plants engaged in more irregular movements than uninfested plants, more *C*. *japonica* tendrils failed to touch infested plants.

### Proximate factors affecting *C*. *japonica* tendril avoidance of infested plants

We first explored whether SMIPVs produced by bean plants caused *C*. *japonica* tendrils to avoid infested plants. We clipped 10 true leaves (*i*.*e*. 30 leaflets) heavily infested by spider mites (>30 adult females and individuals at other stages per leaflet) and placed them in a flask (an odour source). Similar to attached leaves, detached bean leaves produce SMIPVs^15^ and are detected by mites that predate spider mites^16^. Using a pump (GEX e-Air 1000SB; GEX, Osaka, Japan) connected to a transformer, we pumped air (0.3 L/min) containing the volatiles into a transparent flexible straw (5 mm in diameter) bearing 40 vent holes (1 mm in diameter) within a 10-mm length (Fig. 1b). We initially confirmed that an airflow of 0.3 L*/*min did not disturb the coiling response of *C*. *japonica*. We sealed all joints and the tip of the straw with Parafilm M (Parafilm M; American National Can, Chicago, IL) to ensure that the air exited via the vent holes. We then fixed the section bearing the air holes to the future position of tendril movement, as described above. Thus, the SMIPVs emanated from the contact surface. We then observed the coiling responses of *C*. *japonica* tendrils (n = 12) as described above. We used plain air as a control (n = 10).

We also explored whether mite webs caused *C*. *japonica* tendrils to avoid infested plants. We placed webs on the surfaces of disposable hinoki cypress chopsticks. The top 3 cm of each chopstick was covered with webs that were <7 days old and collected from heavily infested plants. We clamped the chopsticks with the web-covered portions in the position of future tendril movement (Fig. 1c), and observed the coiling responses (n = 7). Chopsticks lacking webs served as controls (n = 8). We also covered the top 3-cm lengths of chopsticks with webs of the silk spider (*Nephila clavata*), a common predator inhabiting *C*. *japonica* (Yano, personal observation). The webs came from spider nests found on Kyoto University’s campus. Each nest averaged >50 cm in diameter and was sampled only once (n = 8). Tendril coiling was observed as described above and the differences compared using Fisher’s exact test with the Holm-Bonferroni correction for multiple comparisons.

Finally, we explored whether mite intrusion onto *C*. *japonica* tendrils explained the tendril avoidance of infested plants. Mites moved onto tendrils immediately after the tendrils contacted either heavily infested plants or mite webs on chopsticks; thus, mite intrusion might induce tendril curling and withdrawal from the target. We inoculated ten female mites onto each of seven tendrils, the bases of which were grease-banded, simulating the number of mites running onto tendrils during the above experiments. After 1 h, we explored whether the tendrils had curled and compared the proportion of curled tendrils to that of tendrils that curled after contact with mite webs on chopsticks in the previous experiment.

### Ethics

This article does not contain any studies with human participants or animals.

## Results

### Coiling responses of *I*. *nil* and *C*. *japonica* to bean plants infested with spider mites

**Figure 2:**
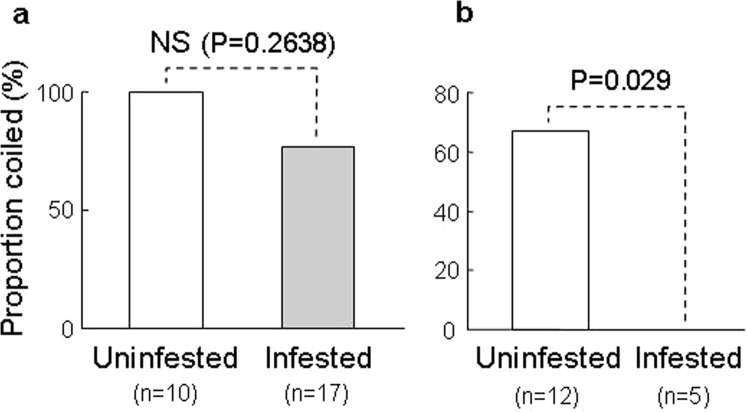
The coiling responses of (**a**) *I. nil* vines and (**b**) *C. japonica* tendrils to spider mite-infested/uninfested bean plants.

The proportions of *I*. *nil* vines that coiled around spider mite-infested and -uninfested bean plants did not differ significantly (*P* = 0.2638, Fisher’s exact test, Fig. 2a), suggesting that *I*. *nil* vines did not discriminate between infested and uninfested plants.

By contrast, the proportion of *C*. *japonica* tendrils that coiled around spider mite-infested plants was significantly lower than the proportion that coiled around uninfested plants (*P* = 0.029, Fisher’s exact test, Fig. 2b), suggesting that *C*. *japonica* tendrils avoided mite-infested plants. After the *C*. *japonica* tendrils touched infested plants, the tendrils curled and withdrew. About ten mites ran onto each tendril during the brief contact, but most soon turned back because the bases of the tendrils were blocked by Tanglefoot.

### Proximate factors affecting *C*. *japonica* tendril avoidance of infested plants

**Figure 3:**
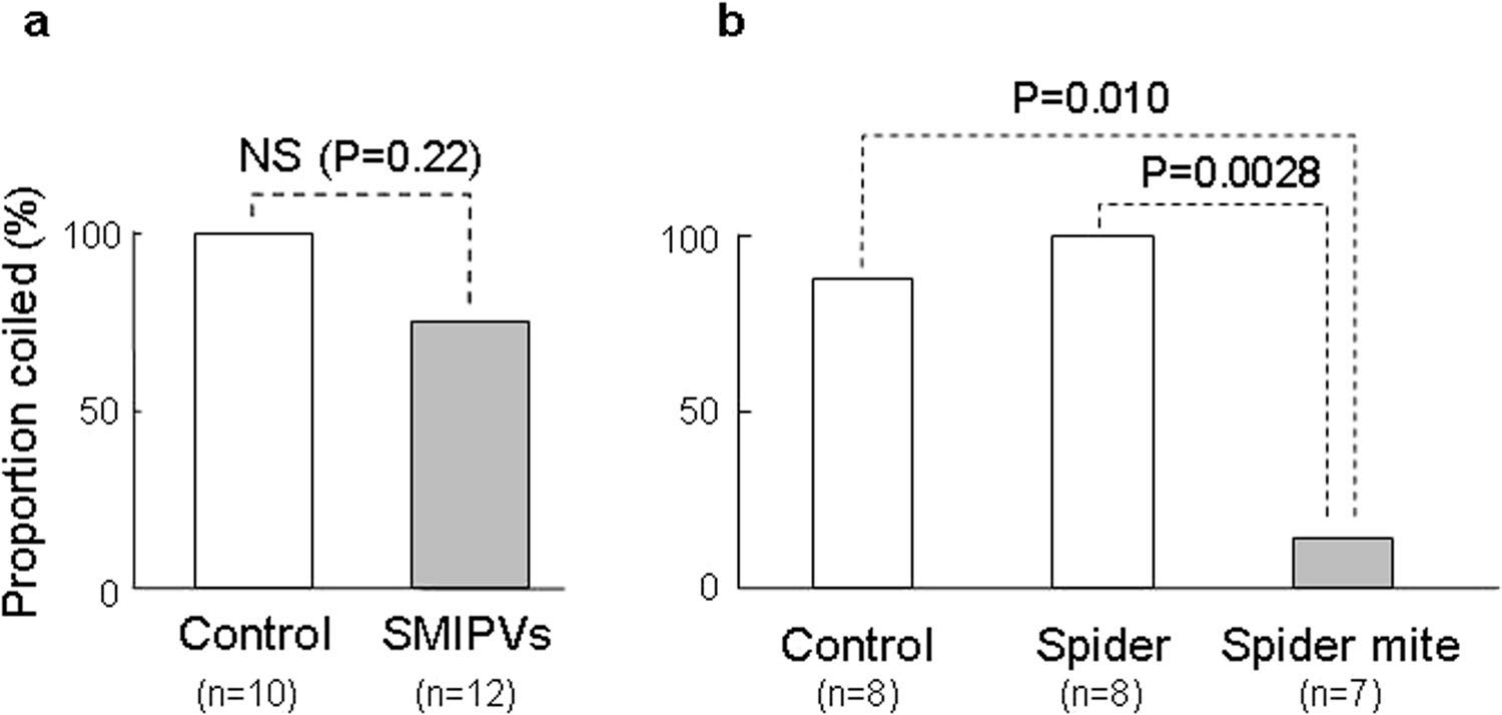
The effects of (**a**) SMIPVs and (**b**) spider mite webs on the coiling responses of *C. japonica* tendrils.

All tendrils coiled around straw sections emitting plain air. A slightly lower proportion of tendrils coiled around sections emitting SMIPVs, but the difference was not significant (*P* = 0.2208, Fig. 3a), suggesting that SMIPVs do not explain why *C*. *japonica* tendrils avoided mite-infested plants. The proportion of tendrils that coiled around chopsticks covered with mite webs was significantly lower than that coiling around control chopsticks (*P* = 0.010, Fisher’s exact test followed by the Holm-Bonferroni correction, Fig. 3b), indicating that the tendrils avoided webs. Moreover, the proportion of tendrils that coiled around chopsticks covered with mite webs was significantly lower than the proportion that coiled around chopsticks covered with spider webs (*P* = 0.0028, Fisher’s exact test followed by the Holm–Bonferroni correction, Fig. 3b), indicating that the tendrils distinguished spider from mite webs. Thus, mite webs may mediate the avoidance of mite-infested plants by *C*. *japonica* tendrils.

**Figure 4:**
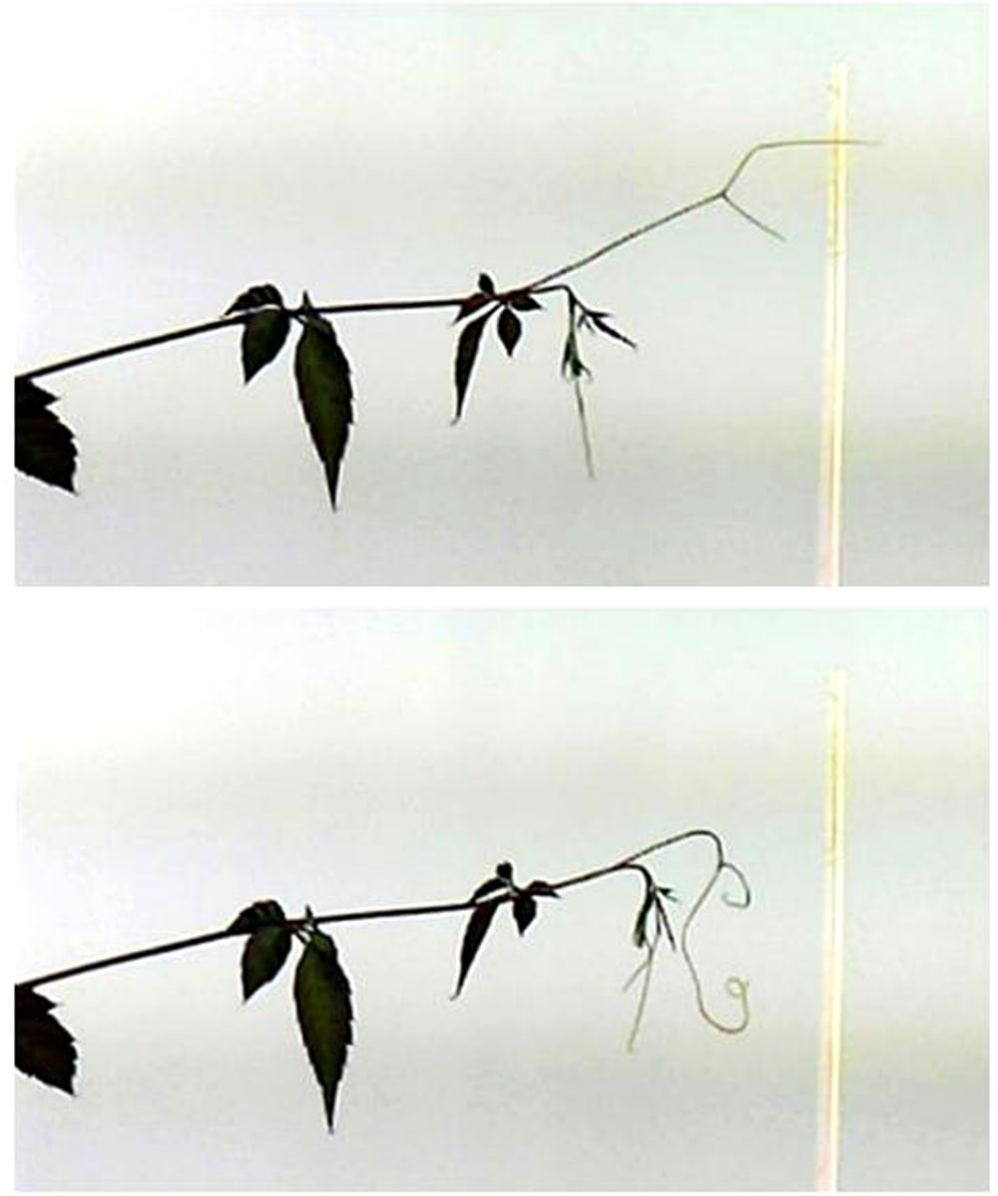
A curling *C. japonica* tendril after contact with spider mite webs on a chopstick.

Six out of seven tendrils that contacted mite webs on chopsticks curled up (Fig. 4); no tendril out of seven curled after mite inoculation (*P* = 0.0047, Fisher’s exact test), suggesting that mite intrusion onto tendrils does not explain why *C*. *japonica* tendrils avoided mite-infested plants. Curled tendrils immediately regained their original shape after they withdrew from the mite webs.

## Discussion

*Cayratia japonica* tendrils avoided coiling around mite-infested plants, preventing “contact infection”. Mites running onto *C*. *japonica* tendrils before tendril withdrawal could seriously damage *C*. *japonica* if the mites then reproduced. However, mites outside their protective webs are readily preyed upon by generalist predators, such as ants and the predatory mite *Euseius sojaensis*^17,18^, which also feed on pearl bodies on the surface of *C*. *japonica*^14^.

By contrast, circumnutating *I*. *nil* vines did not avoid mite-infested plants. As *I*. *nil* (var. Heavenly Blue) is often used as a “green wall”, its ability to indiscriminately coil around all contacted substances may have been artificially selected. Indeed, *C*. *japonica* tendrils seem to be more cautious; some tendrils did not coil around harmless materials such as uninfested bean plants and control chopsticks.

Although SMIPVs emitted by plants become rapidly diluted^19^ or mixed with volatiles emitted by other local infested plants^20^, SMIPVs emitted proximately (*i*.*e*. from contacted plants) might be detectable by plants that produce vines. Thus, we explored whether SMIPVs were involved in the avoidance of mite-infested plants by *C*. *japonica* tendrils, but found no evidence supporting this suggestion.

In fact, mite webs seemed to explain the avoidance. Using webs to indicate the presence of mites is reasonable, as only spider mites (of the many potential herbivores that could feed on *C*. *japonica*) produce webs. Interestingly, *C*. *japonica* tendrils distinguished webs of spider mites from those of the silk spider, which often occurs on *C*. *japonica*, and may protect the plant from certain herbivorous insects, but never damages the plant in the manner of spider mites. Consequently, *C*. *japonica* tendrils would coil around harmless substances bearing spider webs.

*Cayratia japonica* tendrils generally curled up when avoiding mite-infested plants and mite webs. The webs contain many living mites, which indeed ran onto *C*. *japonica* tendrils during the brief contact. However, inoculation of mites onto *C*. *japonica* tendrils did not cause curling, suggesting that curling is not a passive withering caused by mite infestation but rather attributable to some property of the webs (*e*.*g*. contact chemicals). Moreover, curling was reversible; curled tendrils resumed their original shape after withdrawing from mite webs, further suggesting that the curling of *C*. *japonica* tendrils is an active process.

Avoidance of coiling around mite-infested plants may not be restricted to *C*. *japonica*; many plants that produce vines may share this property. Further investigation is needed. Moreover, if the proximate factors explaining the avoidance were known, it would be possible to manufacture fencing materials acceptable to cultivar vines such as morning glory but avoided by wild vines that are sufficiently “clever” to avoid mite webs on contacted plants.

## Supplementary information

Supplementary material

## Data Availability

All data can be found in the Supplementary Material.
